# Evaluation of Vineyard Cropping Systems Using On-Board RGB-Depth Perception

**DOI:** 10.3390/s20236912

**Published:** 2020-12-03

**Authors:** Hugo Moreno, Victor Rueda-Ayala, Angela Ribeiro, Jose Bengochea-Guevara, Juan Lopez, Gerassimos Peteinatos, Constantino Valero, Dionisio Andújar

**Affiliations:** 1 Laboratorio de Propiedades Físicas (LPF_TRAGRALIA), ETSIAAB, Universidad Politécnica de Madrid, 28040 Madrid, Spain; hugo.moreno.parrizas@csic.es (H.M.); constantino.valero@upm.es (C.V.); 2 Centre for Automation and Robotics, CSIC-UPM, Arganda del Rey, 28500 Madrid, Spain; angela.ribeiro@csic.es (A.R.); jose.bengochea@csic.es (J.B.-G.); jlopezcorrea@conicet.gov.ar (J.L.); g.peteinatos@uni-hohenheim.de (G.P.); 3 Norwegian Institute of Bioeconomy Research, NIBIO Særheim, Postvegen 213, 4353 Klepp Stasjon, Norway; victor.rueda.ayala@nibio.no

**Keywords:** depth cameras, Kinect v2, 3D reconstruction, woody crops, vineyards

## Abstract

A non-destructive measuring technique was applied to test major vine geometric traits on measurements collected by a contactless sensor. Three-dimensional optical sensors have evolved over the past decade, and these advancements may be useful in improving phenomics technologies for other crops, such as woody perennials. Red, green and blue-depth (RGB-D) cameras, namely Microsoft Kinect, have a significant influence on recent computer vision and robotics research. In this experiment an adaptable mobile platform was used for the acquisition of depth images for the non-destructive assessment of branch volume (pruning weight) and related to grape yield in vineyard crops. Vineyard yield prediction provides useful insights about the anticipated yield to the winegrower, guiding strategic decisions to accomplish optimal quantity and efficiency, and supporting the winegrower with decision-making. A Kinect v2 system on-board to an on-ground electric vehicle was capable of producing precise 3D point clouds of vine rows under six different management cropping systems. The generated models demonstrated strong consistency between 3D images and vine structures from the actual physical parameters when average values were calculated. Correlations of Kinect branch volume with pruning weight (dry biomass) resulted in high coefficients of determination (R^2^ = 0.80). In the study of vineyard yield correlations, the measured volume was found to have a good power law relationship (R^2^ = 0.87). However due to low capability of most depth cameras to properly build 3-D shapes of small details the results for each treatment when calculated separately were not consistent. Nonetheless, Kinect v2 has a tremendous potential as a 3D sensor in agricultural applications for proximal sensing operations, benefiting from its high frame rate, low price in comparison with other depth cameras, and high robustness.

## 1. Introduction

Accurate reconstruction of plant geometrical features is of crucial importance for decision making processes aiming at optimal agricultural crop management. Reconstructing plant shape and acting according to the plant status enable farmers to improve crop yield, while reducing agrochemicals and operational costs. Plant shape reconstruction mainly requires accurate knowledge of its structural parameters, which are closely linked to final yield and biomass [1]. In this regard, canopy volume and foliage are related to plant parameters, such as LA (leaf area) and LAD (leaf-area density). These parameters are extremely important, since they are directly linked with the photosynthesis and transpiration processes [2].

The latest breakthroughs in technological developments have widened a horizon of possibilities; nowadays, novel sensing techniques can properly accomplish assessment of crop, weeds or plant diseases. Therefore, new approaches for agricultural applications to optimize workflows and minimize manual labour are arising [3]. Practical use of sensing systems changes the paradigm of traditional agriculture, particularly agricultural research. Conventional practices, deeply ingrained in most farmers, involve manual procedures with destructive sampling [4,5], which often lead to wrong decision-making for agronomic management [6]. Thus, 3D digital models using novel sensing technologies can reduce labour cost, increase sampling areas with higher accuracy, while increasing yield and reducing inputs through the use of site-specific applications.

Over recent years, manifold optical non-destructive sensors have been widely used for precision agriculture (PA) tasks, facilitating the development of fast, non-invasive and highly accurate plant reconstruction methods. A wide variety of sensors, from budget systems such as ultrasonic [7] to expensive multispectral or fluorescence sensor systems, have been researched. Although the aforementioned systems provide several plant parameters, their accuracy is low when dealing with vineyard reconstruction in some applications. The calculated volume is only based on the external layer while 3D models created by light detection and ranging (LiDAR) or red, green and blue-depth (RGB-D) cameras calculate the internal volume since they are able to reconstruct the empty internal space. Laser scanners offer three-dimensional with adequate accuracy and high-density measurements of plant geometric characteristics. LiDAR systems can reconstruct plant shape in order to estimate key architectural parameters such as crop height, crop width, crop volume or leaf area. Ground-based sensors have the potential to achieve multiple high-resolution datasets at reasonable cost and are remarkably efficient for range measurements using agricultural machinery [5]. LiDAR or similar distance-measurement systems can be easily mounted onto current machinery to scan crops while doing other agronomical tasks. In regard to vineyards, a LiDAR sensor combined with a Global Positioning System (GPS) receiver installed on top of a tractor, was applied to analyse the distribution of the vine canopy on different grape varieties in order to determine the most suitable rate spraying volume [8]. Even though LiDAR sensors can reconstruct vineyard plants with high accuracy, colour information is missing. This RGB information can be crucial on certain processes for object separation between ground, vegetation, grapes or any other object in different colours in the scene.

RGB cameras have been the most common optical sensor and widely used to assess phenotypic traits for several purposes. Some approaches have differentiated vegetation from vineyard crops. For example, automatic methods were combined for extracting different vegetation indices and elevation models [9]. RGB information is obtained from ground-based and aerial visible imaging data. The acquired images are analysed using algorithms able to segment the set of RGB channels that have been proposed for quick and simple description of plant growing dynamics [10]. However, data assessment from RGB images is sometimes limited due to constraints, such as leaf overlapping or plant shadows, which make difficult a correct assessment of important plant parameters [11]. RGB data can be processed into three-dimensional models using photogrammetry procedures. Andújar, et al. [12] adapted low-cost techniques, structure from motion (SfM) and multiview stereo (MVS) to create 3D models and reconstruct plants from single RGB images. Botterill, et al. [13] designed a prototype robot for the automatic pruning of grape vines with trinocular stereo cameras as it moves. A computer vision system creates a three-dimensional (3D) vine model, an artificial intelligence (AI) system determines which canes to prune, and a six-degree robot arm makes the necessary cuts. Similarly, the architecture of leafless trees in field settings was assessed using a high accuracy industrial robot aided with two colour cameras mounted on the end-effector through segment target regions in images to determine tree structure autonomously [14]. Similarly, 3D models can also be built using the RGB information acquired through remote-sensing systems. This was the case for a vineyard crop were colour model imagery acquired with an unmanned aerial vehicle was used to describe the 3D macro-structure [15]. Amongst RGB systems, depth cameras (RGB-D) such as the Microsoft Kinect sensor (Redmond, WA, USA), due to their distinctive appealing characteristics [16] have received widespread approval in precision agriculture [17,18]. Kinect v2 in 2014, was initially conceived as commercial videogame controller device for the Xbox game console [19]. Due to their popularity in the market this RGB-D camera has attracted the attention of many researches to apply its functioning principle to a wide array of fields beyond video gaming [20]. Interestingly Vit and Shani [18] measured maize stems width using four different RGB-D cameras: Microsoft Kinect v2, Orbbec Astra S, Intel SR 300, and Intel D435. They concluded that Intel D435 is more suitable for outdoor agricultural-oriented tasks. Kinect is a motion device that enables users to have contactless interaction with computers and game consoles mainly through gestures. The first version (Kinect v1) was commonly used indoors, for instance with applications in greenhouses. Kinect v1 is based on a structure light triangulation method, i.e., the projection of a speckle pattern for depth sensing. Unlike its predecessor, the second version (Kinect v2) works on the basis of the time of flight (ToF) concept, being more accurate and precise when detecting depth [21]. Although the Kinect sensors were designed to work indoors under controlled and particularly low light conditions [22] Kinect v2 seems to be less affected by daylight [23]. Andújar, et al. [24] assessed the potential of the Kinect v2 sensor to characterize two trees species of contrasting architecture under different wind conditions. The authors established a wind speed limit of 18 km∙h-1 as the threshold for good estimations of height and LA, as a major determinant of nutrient application. Further tests of the Kinect v2 have been carried out on woody crops. Bengochea-Guevara, et al. [25] developed a mobile platform to facilitate the use of 3D modelling capabilities in this sensor. They concluded that the system was sufficiently accurate for assessments on large areas, at different times of the year and under uncontrolled daytime light. Therefore, assessment of key geometric characteristics, i.e., phenotypic traits, using contactless optical technologies can be achieved on the whole cropped field, which aid in selecting the best strategies to improve crop yield. In addition, the use of non-destructive methods prior to cropping operations is necessary for better decision-making procedures. The use of novel technologies to improve the agronomic management of fields can reduce managing costs while maximizing yield and reducing environmental impact. Regarding vineyard crops, it is of great importance due to its economic impact. The use of sensing devices can substantially improve the accuracy of agronomic tasks such as pruning or fertilization. The use of more accurate methods based on crop sensing and reconstruction would allow information to be subtracted from 3D models to improve decision-making processes. The estimation of branch volume and structural information can improve pruning systems which would positively impact crop yield and crop management. Additionally, the automatic estimation of branch volume and weight can be a good indicator of those branches that need to be pruned and to figure out the most appropriate timing for pruning. Since grapes are one of the world’s most valuable horticultural crops, covering 75,000 km^2^ worldwide [26] the use of automatic systems for decision making are in great demand. Over the past two decades, these systems have been used extensively within the discipline of precision viticulture [27], thus benefiting advancement and breakthroughs in site-specific management studies on vineyards [28]. Measuring wood volumes in perennial crops such as vineyards and orchards is an important index for the evaluation of biomass production, carbon storage, and cycle. The counting and weighing of wood (canes) at the age of one year during pruning is the most interesting and insightful way of showing vine balance, extensively utilized in terms of management purposes by grape growers [29]. Many vine species are pruned by hand, and this is often the most labour-intensive and expensive task in the vineyard [30]. Since dormant pruning must be carried out during the winter months, it means they represent arduous work under often harsh weather conditions. Measuring wood volumes in perennial crops such as vineyards and orchards is an important index for the evaluation of biomass production, carbon storage, and cycle. The counting and weighing of the wood (canes) of the age of one year during pruning is the most interesting and insightful way of showing vine balance, extensively utilized in terms of management purposes by grape growers [29]. Many vine species are pruned by hand, and this is often the most labour-intensive and expensive task in the vineyard [30]. Since dormant pruning must be carried out during the winter months, it means they represent arduous work under often harsh weather conditions. In this study, a non-destructive measuring technique was implemented to assess major geometric traits of vines (individual plants) on measurements recorded by a Kinect V2 mounted on a mobile platform. The overall goal in this work was to verify the performance of RGB-D cameras as a reliable system to reconstruct the 3D architecture of vines. This 3D architecture should correspond to different management practices, while its relationships with future yield and pruning remain. The 3D models were used to determine branch volume, then compared with ground truth values i.e., vineshoot weight and yield. This non-invasive methodology was expected to improve the assessment approach in order to generate detailed morphological parameters of vines comparable with real plant values.

## 2. Materials and Methods

### 2.1. Site Location

The experiment was carried out in a 2 ha vineyard field located at the research station of “El Socorro” (IMIDRA, Colmenar de Oreja, Madrid, Spain, 40°8′N, 3°22′W). Data collection was undertaken during winter 2018 during three consecutive days. The climate is characterized as Mediterranean Continental, i.e., cold winters and hot summers with low precipitation (over 400 mm/year). The field was divided into six management treatments according to a variety of tillage and herbicide application systems. In addition, management cropping systems varied in order to study the influence of those different techniques on final yield. The rest of the applications were kept constant. Fertilization, pesticide treatments and pruning were the same for the whole field and the experimental plots. Every type of management area was replicated four times. Thus, the whole studied field was divided into six different cultivating techniques, and each of them had four replications of three rows, i.e., replications, in different locations within the same vineyard. Each sample consisted of 10-vine batches according to different ways of cultivating: (a) Traditional system with intensive agronomic pressure using three harrowing treatments and herbicide treatments based on inter-row treatment with glyphosate. (b) Low-input or mixed systems combining treatments with pesticides (herbicides and fungicides) and mechanical work. The inter-row area received a harrow operation and mechanical weed control between vineyards using an in-row weeder. (c) Mechanical weed control (using an in-row weeder) in combination with field rotation of vegetation cover every three years using natural coverage without fertilization. The last rotation began in 2015, at the beginning of the series under study. (d) Same treatment as in (c) but including fertilization. (e) Harrowing treatment in the inter-row area. (f) The vegetation cover was left in the inter-row areas and intra-row harrowing. Every treatment was scanned during January 2018. The monitored plots were manually pruned on 20 and 21 February. The remaining sheaves per plot were identified and collected and those branches were taken to the laboratory for dry biomass determination. The branches corresponding to each plot were separately dried for 48 h at 80 °C in order to weigh the dry biomass. Once the samples were dried, they were weighed. The values of dry biomass obtained were used for comparison with those volume values obtained from the 3D model. 

### 2.2. Sampling System

**Figure 1:**
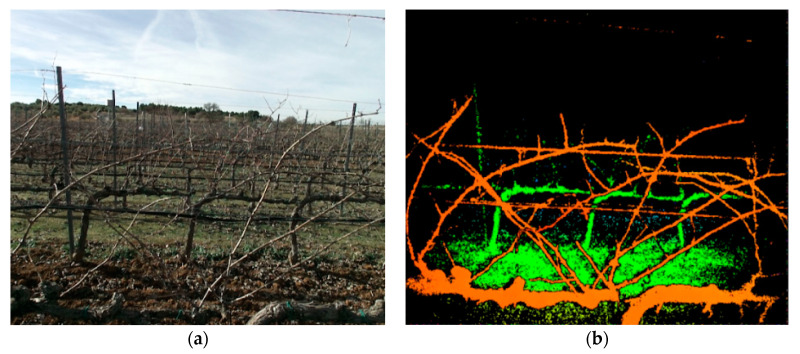
Images supplied by the Kinect v2 sensor. (**a**) Corresponds to the red, green and blue (RGB) channel. (**b**) RGB depth image in a false colour scale where orangeish colours represent the foreground and greenish colours the background vines architecture.

A self-developed electric mobile platform was used for data acquisition. The vehicle travelled in a straight line, parallel to the vine row and in the centre of the inter-row area. Sensors were installed on a height-adjustable bar in front of the platform. This platform is based on a compact commercial electric car, Twizy Urban model (Renault, Boulogne-Billancourt, France), with a compact dimension suitable for sampling most crops, also adapted to vineyard conditions. The vehicle dimensions are 2.32 m length, 1.19 m width, and 1.46 m height, and the unladen weight is of 450 kg. The platform incorporates a 13 kW electric motor which allows sampling at 5 km/h, producing insignificant vibrations at that speed. This sampling speed ensures acquisition of high-quality information for 3D data modelling. This sampling system was equipped with the necessary instrumentation for data collection and remote operation mode. A Microsoft Kinect v2 RGB-D sensor-based system was set up for data acquisition. The Microsoft Kinect v2 supplies RGB images (1920 × 1080 pixels) together with depth information (512 × 424 pixels), up to 30 fps. Figure 1 shows the same recorded section of the vine row in the vineyard during the tests. 

**Figure 2:**
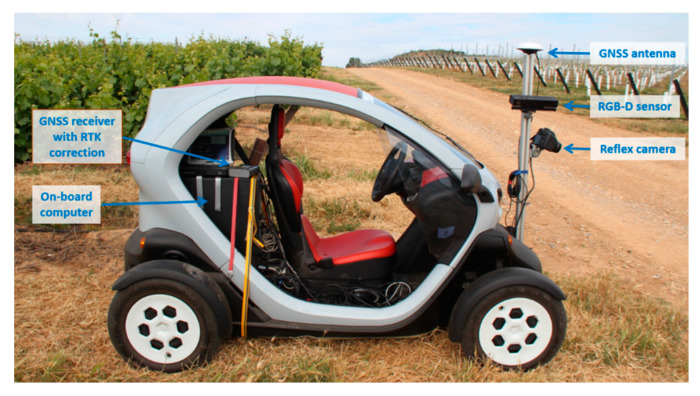
Electric platform equipped with an on-board computer for autonomous navigation and signal processing. The Kinect v2 sensor was installed on an adjustable height structure, provides RBG and distance information associated to Global Positioning System (GPS) coordinates.

The functioning range of the sensor fulfills the inspection criteria of the vineyard rows, while the non-interesting items, such as those that are exceptionally near and those in remote areas that usually include other vineyard rows, are avoided. The time of flight (ToF) principle is used for depth data acquisition. This system is included inside the sensor, which calculates the distances according to the time that a light pulse takes to travel from the light source to the object and back to the sensor. The operating principle is as follows: the distance to be measured is proportional to the time required for the light to travel from the infrared (IR) laser diode (i.e., the emitter), back and forth to the target (i.e., the impacted object). The reflected beam is collected by the depth sensor. The phase shift between the source of light and the bounced light is used to calculate the depth at pixel level, assigning a distance value to each pixel. Thus, high-resolution depth maps can be acquired by measuring every single pixel of the nearest objects. The field of view (FOV) for depth acquisition is 70 degrees horizontally and 60 degrees vertically, with a range from 0.5 m to 4.5 m, being lower when working outdoors (Fankhauser et al., 2015). The Kinect v2 sensor was connected to an on-board computer (Intel Core i7-4771@3.5GHz processor, 16 GB of RAM, NVIDIA GeForce GTX 660 graphic card). The Kinect v2 sensor was installed on the aluminium support structure to obtain the lateral projection of the vines (Figure 2).

### 2.3. Data Processing

The acquired information was processed following the procedure described by [31]. The algorithm reconstructs large regions using the fusion of different overlapped depth images, storing information only on the voxels closest to the detected object [32]. Since the camera position is known, the following input images that arrive are properly located using the ray-casting method [33]. The method determines the voxels related to the depth information. This information is used to estimate the position and orientation of the camera. A ray is projected from the camera focus for each pixel of the input depth image to define the voxels in the 3D world that cross each ray. A modified version of the iterative closest point (ICP) algorithm [34] was set up to estimate the position and orientation of the Kinect sensor, allowing six degrees of freedom from the previous information of an image to the subsequent input image. This procedure used a desktop computer (Intel Core i7-6900K@3.2 GHz processor, 64 GB of RAM, NVIDIA GeForce GTX Titan X graphics card) to generate the 3D models of sampled vineyard plots. Then, an automatic data filtering process to remove isolated points was applied. Points of an average distance (average of 64 nearest neighbours) greater than the standard deviation distance to all neighbouring points were considered outliers. This calculation process simplified the 3D model representation, and from these models, plant volume could be estimated for each sampled plot. 

**Figure 3:**
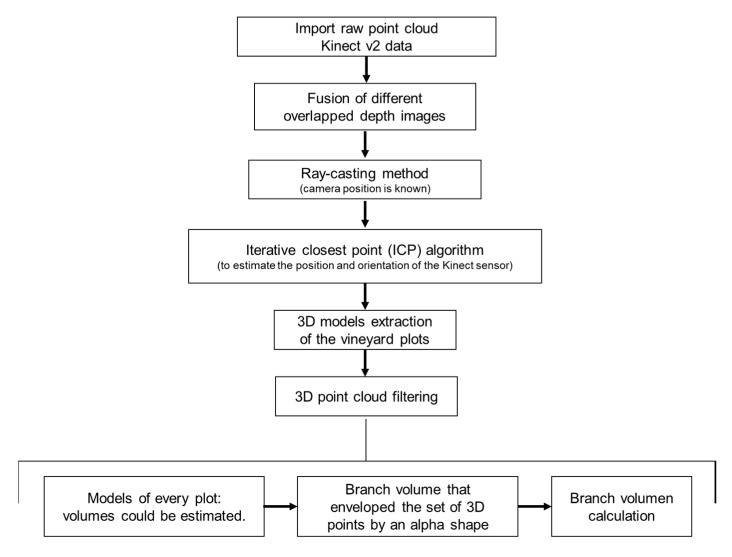
Data-processing block diagram.

The first step allowed estimation of the volume wrapping the set of 3D points by an alpha shape [35]. The alpha-shape represents the outline that surrounds a set of 3D points [11,25,36] and specifies how tight the body fits the points. The index α defines the degree of fitness. The more the α value decreases, the tighter the outline fits the points, producing a smaller surface to enclose a set of 3D points. In the present study, the α enclosing the smallest volume whilst maintaining a solid surface free from voids was sought. The entire process can be seen in Figure 3.

**Figure 4:**
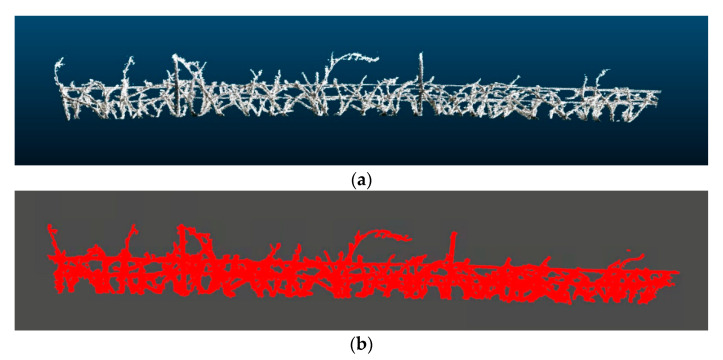
(**a**) Example of 3D reconstruction of a scanned point cloud of a vineyard row after outlier filtering. (**b**) Alpha shape of the point cloud showed in (**a**) with alpha = 0.1 that represents the outline that surrounds a set of 3D points.

Various analyses were carried out with different values of α, showing that the α-shape produced using 0.1 was the most appropriate to the actual outline of the vineshoots (Figure 4). For this purpose, the R package alphashape3d [37] was used for determination of α. The final branch volume was calculated using the same library.

### 2.4. Statistical Analysis

Regression analyses were used to evaluate the system’s capability to quantify the weight of pruning remains from 3D models and its relationship with different cropping systems. In addition, the relation between those cropping systems was compared with grape yield. The cases showing a positive correlation between the observed vineyard yield and the measured vineyard dry biomass were taken as meaningful, and compared with the corresponding acquired sensor data. The information obtained from the 3D models was processed and statistically compared with branch volume as a key feature of actual pruning remains weight and final yield. The pruning remains (actual ground truth parameters) were weighed, then compared with the volume estimated from the 3D models. A predefined linear approach was used to quantify the geometrical reliability of the Kinect v2 sensor for estimating branch volume and yield. The linear regression models were applied to Kinect v2 volume against vineyard yield, and Kinect v2 volume against branch dry weight. Firstly, averaged data of all treatments and sensor measurements were compared, to verify the general meaningful relationship. Afterwards, further linear models were applied to test whether the agronomic treatments and factor interactions effects causing different slope coefficients. To exclude non-significant factor or factor interaction effects on the regression parameters, F-tests (alpha = 0.05) were performed to reduce the model complexity. Moreover, approximate 95%-confidence intervals were calculated with the Delta method, to facilitate a direct comparison of the various treatments and regression coefficients). These protocols allowed testing the phenotyping capabilities of this procedure under different management systems, including the possibility to detect differences among agronomic treatments. Statistical analysis and plots were carried out using R (R Core Team, 2020) version 4.0.3 “Bunny-Wunnies Freak Out” and the package ‘ggplot2’ [38].

## 3. Results and Discussion

**Figure 5:**
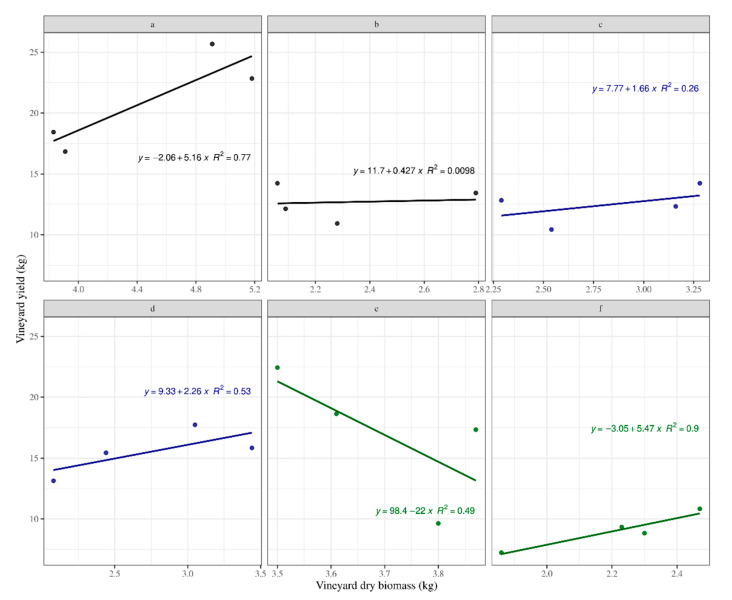
Behaviour of the measured (truth) vineyard dry biomass versus the measured (truth) vineyard yield, according to the different agronomic treatments (averaged per treatment).

**Figure 6:**
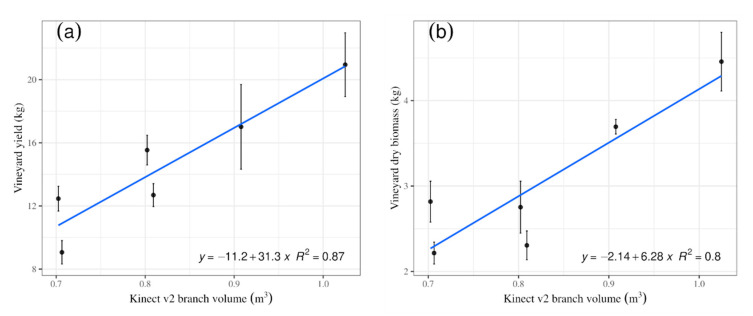
(**a**) Kinect volume versus vineyard dry biomass (average per treatment). (**b**) Kinect volume versus value for grape yield (average per treatment).

The ground management affected in a different manner the relationship with the studied parameters. This difference was not directly perceptible by the human eye. Thus, the use of digital systems is clearly justified. The measured vineyard yield versus the measured vineyard yield were explained by the linear models (Figure 5), indicating differences in slope and intercepts for all treatments. However, treatment ‘e’ showed a negative correlation, which implied inconsistencies in the expected response that the higher the biomass was, the higher the yield should have been. Only treatment ‘a’ showed a statistically significant effect (P = 0.02; Table 1) of the agronomic treatment, with vineyard yield influencing the vineyard dry biomass. In addition, treatment ‘d’ showed a slight tendency, although non-significant (P = 0.07), to be influential. When all data were analysed as a whole, averaging the information by treatment, a significant linear relationship was obtained. The linear model fitted data averaged by treatment for the total Kinect v2 volume well compared with the vineyard dry biomass, indicating that a higher branch volume corresponded to the increasing biomass values with an R2 = 0.80. In addition, a positive linear fit was obtained for the comparison between vineyard yield and Kinect v2 volume (without tutoring prob) with an R2 = 0.87 (Figure 6). 

**Figure 7:**
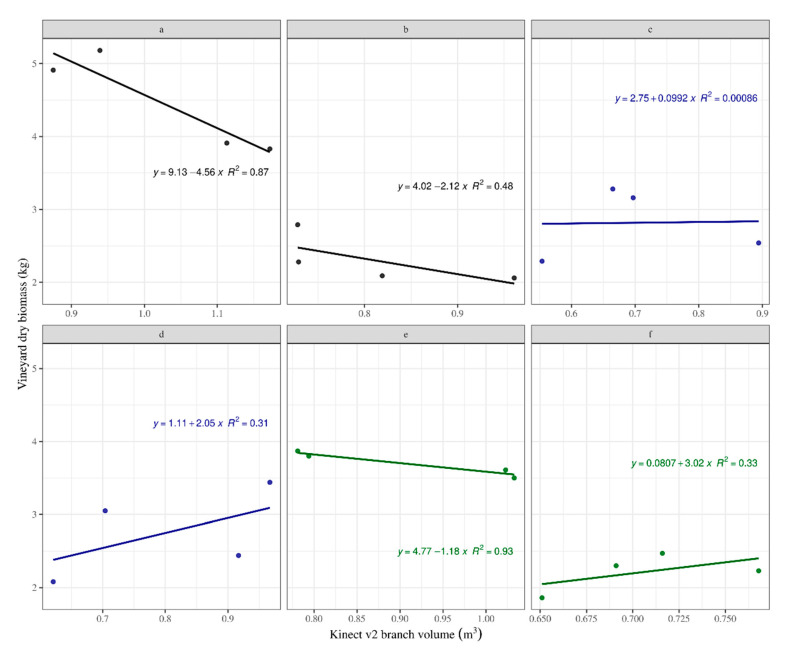
Kinect branch volume versus vineyard dry biomass including effect of treatment.

The linear models including effects of treatments in the regression between Kinect v2 volume and the vineyard dry biomass showed meaningful (positive) and inconsistent (negative) correlations (Figure 7). When the relationships between Kinect v2 volume and measured vineyard yield were compared, considering only the positive correlations, there was no significant difference with the truth data, owing to overlapping 95% confidence intervals of the respective slope coefficients (Table 1). This observation indicates the system’s capability to detect differences and discriminate the treatments d and f, although with a low accuracy (R^2^ < 0.33; Figure 7). A similar effect could be stated for treatment ‘c’; nevertheless, in this case the correlation is really low (R^2^ < 0.001; Figure 7). However, the number of plots could influence this tendency. A higher number of sampling points, such as a division of the measurement per vineyard instead of a whole plot, may fit the requirements to validate the system. The influence of individual treatments was non-significant (p > 0.1) which can be connected with the hypothesis that increasing the number of distance measurements correlate with increasing vineyard biomass measured in the field [39].

**Figure 8:**
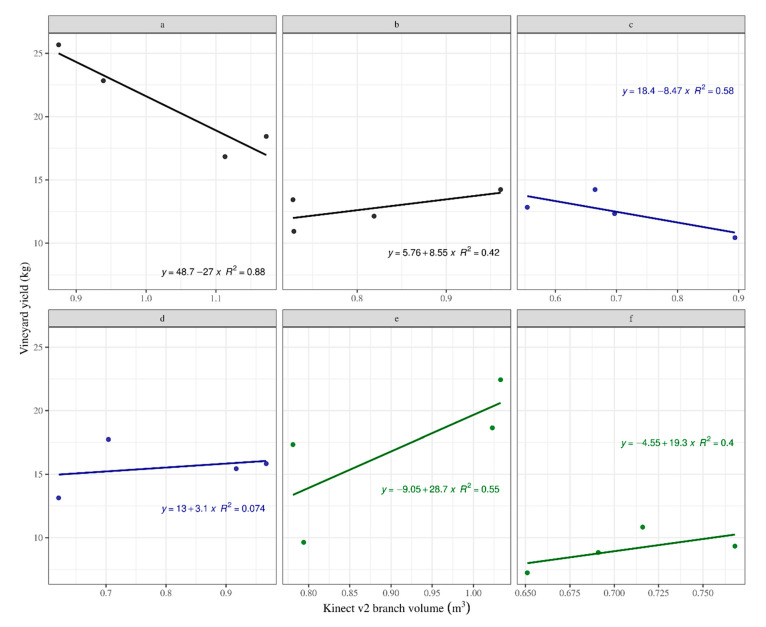
Kinect branch volume versus vineyard yield including the effect of treatment.

A slightly better situation was observed for the linear model between Kinect v2 volume and vineyard yield (Figure 8). In this case, the system showed discriminating capabilities for the treatments b, d, f and even treatment e, with a relatively higher accuracy for treatments b, e and f (0.40 < R^2^ < 0.56). These results support the idea that the system can discriminate 50% of the cultivation systems (i.e., treatments b, e and f), or even more, if results for treatment d are taken into consideration, despite the very low R^2^ (0.07).

Although each treatment was related in a different manner to the ground truth parameters, i.e., dry biomass and vineyard yield, the high concordance in most of the cases proved the accuracy of low-cost sensors for a rapid reconstruction for evaluating pruning volume and its relation with yield. The use of high detailed models is usually demanded for precision operations. The availability of digital information improves decision-making processes when a precision management decision needs to be taken. The majority of branches that should be pruned are of low diameter and most low-cost sensors are not able to reconstruct end-details. The proposed approach was available in the market for gaming. Three-dimensional LiDAR could be an alternative in terms of accuracy and robustness; however, this system is fairly costly [40]. However, the accuracy of depth sensors is sometimes low for reconstruction of end details but less expensive. The use of other systems such as depth cameras or low-cost photogrammetry methods are not able to capture such small details [23]. Furthermore, the fact that Kinect v2 depth measurement quality is poorer at longer distances, where usually end parts of the vines have a small diameter, could explain the discordant agreement when each treatment is assessed separately. Also, the system might struggle to model the tangled network of overlapping vine shoots, worsening the correspondent 3D reconstruction. Thus, small branches are not properly reconstructed as a result when the statistical analysis was performed for each separate treatment and the results were not consistent in some cases, showing subhorizontal slopes.

Although Kinect v2 shows low sensitivity to the lighting conditions [18], since the measurements were carried out around mid-day and sunlight is strongest, it could lead to a slight decrease in performance worsening the proper reconstruction of end details of the vines. The unsuccessful reconstruction of the end information of the branches in direct strong sunlight, could be explained by the standard deviation of the depth data of Kinect V2 for the sensor-to-target distance as reported by Fankhauser, et al. [41] thus generating more point cloud’s invalid points, contributing to detection failures. The previous version v1, at increasing distances, its precision and accuracy were lower. By comparison, Kinect v2 accuracy is almost constant over various distances, while precision also decreases. This could result in some inconsistencies over the separated treatment analysed, which could be minimized by lowering the speed of the vehicle, hence acquiring more redundant data, allowing the fusing of subsequent depth images. A feasible solution at a cost of increasing the budget would be utilizing expensive 3D scanners available in the market offering dense point clouds with mesh resolution of up to 0.1 mm and point accuracy of up to 0.05 mm [13]. Another approach would be possible by sensor fusion data from low-cost 2D laser scanners to digitize three-dimensional crop geometry in the form of point clouds more accurately for the thinner parts of the wood. In this regard, shape-from-silhouette methods work well in the imaging of fine branching structures, and are easily segmentable from the background as demonstrated by Kumar, et al. [42] when reconstructing 3D models of wheat, grass, and a lavender shoot.

Furthermore, other constrains such as variations in temperature or differences in colours with different reflectivity might make depth measurements less reliable [43]. This fact was minimised since during the field trials the vines studied were defoliated; thus, just woody parts were present, showing a homogeneous scene colour. 

## 4. Conclusions

An automatic measurement system for vineyards based on Kinect v2 sensor was assessed. A non-destructive 3D measurement device was designed using a low-cost sensor, scanning and data analysis processes operating automatically by using suitable algorithms. The system is capable of reconstructing 3-D vine shapes in order to assess branch volume. The experiments were carried out in six different management cropping systems, i.e., six different cultivating techniques, during wintertime when vines were defoliated. The result shows that the branch volume measured by the proposed system has a good linear relationship with the reference measurements (i.e., pruning weight), for dry biomass and vineyard yield when average values were taken into account. However, when statistical analysis was performed per each separate treatment the behaviour was not as expected. Although the proposed system can obtain fine 3D vine reconstructions, its inability to measure thin structures i.e., end parts of the vineshoots less than 1 cm in diameter, might explain this mismatch. Further work may include scanning both sides of the vines and adding other sensors to the system to obtain more complete and robust information so that fine structures are not lost. 

## Figures and Tables

**Table 1:** Regression slope coefficients, standard error, P-value of the F-tests and corresponding 95% confidence intervals (95% CI) of the estimated slopes, indicating the meaningful correlations between Kinect v2 calculated volume and the measured response variables vineyard yield and dry biomass.

Response	Regressor	Treatment	Estimate	Std. Error	*p* Value	Lower 95% CI	Upper 95% CI
Vineyard dry biomass	Kinect volume	c	0.10	1.64	0.95	−3.48	3.68
		d	2.05	1.40	0.17	−1.00	5.11
		f	3.02	4.76	0.54	−7.34	13.38
Vineyard yield	Kinect volume	b	8.55	12.26	0.50	−18.15	35.26
		d	3.10	8.10	0.71	−14.54	20.75
		e	28.71	9.68	0.01	7.62	49.80
		f	19.26	27.48	0.50	−40.61	79.13
Vineyard dry biomass	Vineyard yield	a	0.15	0.05	0.02	0.03	0.27
		b	0.02	0.15	0.88	−0.31	0.36
		c	0.15	0.14	0.30	−0.15	0.46
		d	0.23	0.12	0.07	−0.02	0.49
		f	0.16	0.15	0.29	−0.16	0.49

## References

[1] Wang X., Singh D., Marla S., Morris G., Poland J. (2018). Field-based high-throughput phenotyping of plant height in sorghum using different sensing technologies. Plant Methods.

[2] Wu D., Phinn S., Johansen K., Robson A., Muir J., Searle C. (2018). Estimating Changes in Leaf Area, Leaf Area Density, and Vertical Leaf Area Profile for Mango, Avocado, and Macadamia Tree Crowns Using Terrestrial Laser Scanning. Remote Sens..

[3] Kragh M.F., Christiansen P., Laursen M.S., Larsen M., Steen K.A., Green O., Karstoft H., Jørgensen R.N. (2017). FieldSAFE: Dataset for Obstacle Detection in Agriculture. Sensors.

[4] Paulus S., Schumann H., Kuhlmann H., Léon J. (2014). High-precision laser scanning system for capturing 3D plant architecture and analysing growth of cereal plants. Biosyst. Eng..

[5] Rosell Polo J.R., Sanz R., Llorens J., Arnó J., Escolà A., Ribes-Dasi M., Masip J., Camp F., Gràcia F., Solanelles F. (2009). A tractor-mounted scanning LIDAR for the non-destructive measurement of vegetative volume and surface area of tree-row plantations: A comparison with conventional destructive measurements. Biosyst. Eng..

[6] Jimenez-Berni J.A., Deery D.M., Rozas-Larraondo P., Condon A.T.G., Rebetzke G.J., James R.A., Bovill W.D., Furbank R.T., Sirault X.R.R. (2018). High Throughput Determination of Plant Height, Ground Cover, and Above-Ground Biomass in Wheat with LiDAR. Front. Plant Sci..

[7] Andújar D., Weis M., Gerhards R. (2012). An Ultrasonic System for Weed Detection in Cereal Crops. Sensors.

[8] Llorens J., Gil E., Llop J., Queraltó M. (2011). Georeferenced LiDAR 3D Vine Plantation Map Generation. Sensors.

[9] Pádua L., Marques P., Hruška J., Adão T., Bessa J., Sousa A., Peres E., Morais R., Sousa J.J. (2018). Vineyard properties extraction combining UAS-based RGB imagery with elevation data. Int. J. Remote Sens..

[10] Peteinatos G.G., Weis M., Andújar D., Rueda Ayala V., Gerhards R. (2014). Potential use of ground-based sensor technologies for weed detection. Pest. Manag. Sci..

[11] Rueda-Ayala V.P., Peña J.M., Höglind M., Bengochea-Guevara J.M., Andújar D. (2019). Comparing UAV-Based Technologies and RGB-D Reconstruction Methods for Plant Height and Biomass Monitoring on Grass Ley. Sensors.

[12] Andújar D., Ribeiro A., Fernández-Quintanilla C., Dorado J. (2016). Using depth cameras to extract structural parameters to assess the growth state and yield of cauliflower crops. Comput. Electron. Agric..

[13] Botterill T., Paulin S., Green R., Williams S., Lin J., Saxton V., Mills S., Chen X., Corbett-Davies S. (2017). A Robot System for Pruning Grape Vines. J. Field Robot..

[14] Tabb A., Medeiros H. A Robotic Vision System to Measure Tree Traits. Proceedings of the 2017 IEEE/RSJ International Conference on Intelligent Robots and Systems (IROS).

[15] Weiss M., Baret F. (2017). Using 3D Point Clouds Derived from UAV RGB Imagery to Describe Vineyard 3D Macro-Structure. Remote Sens..

[16] Vázquez-Arellano M., Reiser D., Paraforos D.S., Garrido-Izard M., Burce M.E.C., Griepentrog H.W. (2018). 3D reconstruction of maize plants using a time-of-flight camera. Comput. Electron. Agric..

[17] Rosell-Polo J.R., Gregorio E., Gené J., Llorens J., Torrent X., Arnó J., Escolà A. (2017). Kinect v2 Sensor-Based Mobile Terrestrial Laser Scanner for Agricultural Outdoor Applications. IEEE/ASME Trans. Mechatron..

[18] Vit A., Shani G. (2018). Comparing RGB-D Sensors for Close Range Outdoor Agricultural Phenotyping. Sensors.

[19] Pagliari D., Pinto L. (2015). Calibration of Kinect for Xbox One and Comparison between the Two Generations of Microsoft Sensors. Sensors.

[20] Lun R., Zhao W. (2015). A Survey of Applications and Human Motion Recognition with Microsoft Kinect. Int. J. Pattern Recognit. Artif. Intell..

[21] Guzsvinecz T., Szucs V., Sik-Lanyi C. (2019). Suitability of the Kinect Sensor and Leap Motion Controller—A Literature Review. Sensors.

[22] Rosell-Polo J.R., Auat Cheein F., Gregorio E., Andújar D., Puigdomènech L., Masip J., Escolà A., Sparks D.L. (2015). Chapter Three-Advances in Structured Light Sensors Applications in Precision Agriculture and Livestock Farming. Advances in Agronomy.

[23] Lachat E., Macher H., Landes T., Grussenmeyer P. (2015). Assessment and Calibration of a RGB-D Camera (Kinect v2 Sensor) Towards a Potential Use for Close-Range 3D Modeling. Remote Sens..

[24] Andújar D., Dorado J., Bengochea-Guevara J.M., Conesa-Muñoz J., Fernández-Quintanilla C., Ribeiro Á. (2017). Influence of Wind Speed on RGB-D Images in Tree Plantations. Sensors.

[25] Bengochea-Guevara J.M., Andújar D., Sanchez-Sardana F.L., Cantuña K., Ribeiro A. (2018). A Low-Cost Approach to Automatically Obtain Accurate 3D Models of Woody Crops. Sensors.

[26] FAO (2014). Faostat: Crops, National Production. Online. http://faostat.fao.org.

[27] Santesteban L.G. (2019). Precision viticulture and advanced analytics. A short review. Food Chem..

[28] Andújar D., Moreno H., Bengochea-Guevara J.M., de Castro A., Ribeiro A. (2019). Aerial imagery or on-ground detection? An economic analysis for vineyard crops. Comput. Electron. Agric..

[29] Tagarakis A., Liakos V., Chatzinikos T., Koundouras S., Fountas S., Gemtos T. (2013). Using Laser Scanner to Map Pruning Wood in Vineyards.

[30] Dryden G. (2014). 2014 Viticulture Monitoring Report.

[31] Nießner M., Zollhöfer M., Izadi S., Stamminger M. (2013). Real-time 3D reconstruction at scale using voxel hashing. ACM Trans. Graph..

[32] Curless B., Levoy M. A volumetric method for building complex models from range images. Proceedings of the 23rd Annual Conference on Computer Graphics and Interactive Techniques, Association for Computing Machinery.

[33] Roth S.D. (1982). Ray casting for modeling solids. Comput. Graph. Image Process..

[34] Chen Y., Medioni G. (1992). Object modelling by registration of multiple range images. Image Vis. Comput..

[35] Edelsbrunner H., Mücke E.P. (1994). Three-dimensional alpha shapes. ACM Trans. Graph..

[36] Colaço A.F., Trevisan R.G., Molin J.P., Rosell-Polo J.R., Escolà A. (2017). A Method to Obtain Orange Crop Geometry Information Using a Mobile Terrestrial Laser Scanner and 3D Modeling. Remote Sens..

[37] Lafarge T., Pateiro-López B. (2017). Alphashape3d: Implementation of the 3D Alpha-Shape for the Reconstruction of 3D Sets from a Point Cloud, 1.3.

[38] The R Foundation R: A Language and Environment for Statistical Computing. https://www.R-project.org/.

[39] Moreno H., Valero C., Bengochea-Guevara J.M., Ribeiro Á., Garrido-Izard M., Andújar D. (2020). On-Ground Vineyard Reconstruction Using a LiDAR-Based Automated System. Sensors.

[40] Jiao J., Yuan L., Tang W., Deng Z., Wu Q. (2017). A Post-Rectification Approach of Depth Images of Kinect v2 for 3D Reconstruction of Indoor Scenes. ISPRS Int. J. Geo-Inf..

[41] Fankhauser P., Bloesch M., Rodriguez D., Kaestner R., Hutter M., Siegwart R. Kinect v2 for mobile robot navigation: Evaluation and modeling. Proceedings of the 2015 International Conference on Advanced Robotics (ICAR).

[42] Kumar P., Cai J., Miklavcic S. (2012). High-throughput 3D modelling of plants for phenotypic analysis. Proceedings of the 27th Conference on Image and Vision Computing New Zealand.

[43] Wasenmüller O., Stricker D. (2017). Comparison of Kinect V1 and V2 Depth Images in Terms of Accuracy and Precision.

